# Operation of Lithotripsy

**Published:** 1842-12-24

**Authors:** F. Campbell Stewart

**Affiliations:** Paris


### MEDICAL EXAMINER.

NEW SERIES.

No. 52.] PHILADELPHIA, DECEMBER 24, 1842. [Vol. I

### CLINICAL REPORTS.

### HÔTEL DIEU, OF PARIS.

*Operation of Lithotripsy.—Breaking of the Instrument in the Bladder—death of the patient.* Reported by F. Campbell Stewart, M. D.

Nicolas Joly, aged 44 years, entered the Hôtel Dieu, at Paris, on the 11th of October, 1842, to be treated for an urinary calculus, with which he had been suffering for several months. He was placed in the wards of Monsieur Blandin, (Salle St. Bernard, No. 45,) and it was decided that his stone should be removed by the process of lithotripsy.

The first operation was performed on the 17th of October, in the presence of Dr. Leroy d'Etiolles, and a number of students; it was some time before the operator could get the calculus between the branches of Heurteloup's *brise pierre fenetrée*, and, when seized, it was found so hard as to require the exercise of very considerable force before it would yield. After breaking off several fragments, however, the instrument was withdrawn, and the patient directed to take a warm bath.

The second operation was performed one week after the first, viz: on the 24th ultimo; and it was on this occasion, that the female branch of the instrument (No. 2—made by Charriére) broke off, at about one inch from its extremity, and fell into the bladder. No attempt was made to extract the broken piece, and it was determined to have recourse to Lithotomy, so soon as the patient should be in a condition to render the success of the operation probable.

I saw the patient on the 27th, three days after the accident; his countenance then expressed great anxiety and uneasiness; he had had two chills; his pulse was full and feverish; he complained of no pain in the hypogastric region; nor indeed anywhere, but in the right side about the liver and kidney; where it was sometimes very great, and always increased by pressure; in a word, he was labouring under nephritis. On this occasion, the orifice of the urethra was enlarged by Monsieur Blandin, and he removed, with a small scoop, a number of fragments of stone, which had been broken off during the last operation, and, following the course of the urine, been arrested in, and behind the fossa navicularis. Between this period and the second of November, I attended the Hôtel Dieu regularly, expecting every day that M. Blandin would cut his patient, and extract the stone and fragment of the instrument. This was not done, however; and, after a daily succession of chills, followed by fever and perspiration, Joly died on the morning of the 3d instant.

The autopsy was made 24 hours after death, and the following was found to be the condition of the organs examined:—

*Bladder*—Hypertrophied, and likewise much contracted. Presents evidence of recent inflammation, and several small fungous tumours about its fundus. Contains a mulberry calculus, (of oxalate of lime,) of the size of a small hen's egg, from the sides of which several pieces have been broken off by the *brise-pierre*. Some of these fragments still remain ; the rest were expelled during life along with the urine. The portion of instrument which was broken off, is found at the bottom of the bladder, and has become quite black.

*Prostate*—Considerably enlarged, but otherwise healthy.

*Urethra*—Perfectly healthy in its whole course, but with some portions excoriated by the fragments of stone, which had to be forcibly extracted.

*Ureters*—In a normal state.

*Kidneys*—Enlarged, and evidently in a state of chronic inflammation. Several small purulent abcesses are found to exist in the cortical substance of the right one.

*Liver*—Hypertrophied, and of a dark green colour throughout.

*Spleen*—Much softened, and likewise enlarged.

*Lungs*—Healthy, with the exception of some ancient adhesions of the pleura, and a large number of very minute collections of pus about the circumference of the base of the right one.

*Brain and Heart*—Not examined.

No traces of peritonitis.

*Remarks.*—In the history of the foregoing case, we have an instance of the repetition of an accident which has already happened more than once ; and, as it is calculated, without explanation, to do serious injury to the cause of lithotripsy, I may be permitted to offer a few extenuating remarks :

The patient, whose case has just been detailed, was at the period of his admission into the hospital, in such a condition as to preclude from the mind of a cautious surgeon, the idea of an immediate operation, either by lithotomy or lithotripsy. He was exceedingly irritable, and, at that time, even complained of constant pain in the regions of the liver and right kidney, proving that inflammation already existed, either in a chronic or incipient stage. If, however, it was judged indispensable to relieve him as soon as possible of his stone, the question arose as to which of the operations would be most applicable to his case ; and here, I think, there was not ground for a moment's hesitation. Lithotomy should undoubtedly have been preferred ; and for the reasons, that the stone was not too large to be removed through the perineum, whilst it was too hard to be easily broken by the *brise-pierre ;* and, in the former case, the source of irritation would have been at once and entirely removed ; whereas, by the latter process, a number of hard, angular pieces of stone would be substituted in place of one large mass, which acting as a constant source of irritation, could but complicate the operation, and thus compromise its success.

Civiale has long since declared his opinion, which he repeated to me a few days since, that the operation of lithotripsy is inapplicable in most cases where the kidneys are diseased. and he is always cautious not to operate until such diseases have been subdued or removed.

Monsieur Blandin's known skill, as an operator, is a sufficient guarantee that the instrument was properly used, although he candidly admits that he employed more force than it was probably safe to resort to.

The instrument itself was one of Charriére's make, and I am assured by him, that it had been fully and faithfully tested with the hardest stones before being delivered at the Hospital.

The only other question, which I think it necessary to examine, is, what influence the accident may have exercised over the issue of the case; and here I am bound to declare, that I cannot conceive how a small piece of smooth, highly-polished steel would be capable of irritating the bladder, or producing consequences more serious, than a large, angular fragment of hard stone, and the more so, when the individual is kept quiet and still in his bed! In conclusion, then, I would attribute, and, I think, justly, the fatal termination of this case to other causes than the influence of the unfortunate accident, to which the result is attributed by some loud declaimers and violent opponents of the ingenious and now, (notwithstanding this accident, and their opposition,) fully established operation of lithotripsy. This must be regarded, then, as one of those unfortunate occurrences which sometimes take place in spite of every precaution, and which it is impossible to forsee.

There is one circumstance connected with the case, which I cannot explain or account for, however; nor do I conceive that it is capable of being justified on any ground. I refer to the delay practised with regard to the performance of lithotomy, after the accident had happened. It appears to me, that the true course, and the one which I should have unhesitatingly adopted, would have been to operate immediately, even within the hour after the accident.

*Paris, November* 11th, 1842.

